# Validation of the Morningness–Eveningness Scale for Children (MESC) with ambulatory circadian monitoring of temperature, light exposure and activity

**DOI:** 10.1111/jsr.14444

**Published:** 2025-01-02

**Authors:** Yaiza Puig‐Navarro, Juan F. Díaz‐Morales

**Affiliations:** ^1^ Individual Differences, Work and Social Psychology Department Complutense University of Madrid; R&D department, Hogrefe TEA Ediciones Madrid Spain; ^2^ Individual Differences, Work and Social Psychology Department Complutense University of Madrid Madrid Spain

**Keywords:** adolescents, circadian parameters, circadian preference, sleep parameters

## Abstract

The external validity of the Morningness–Eveningness Scale for Children was analysed via objective measures of skin temperature, light exposure and motor activity with the ambulatory circadian monitoring methodology. A total of 138 adolescents (57 boys and 81 girls) aged 12–13 years, who in addition to completing the Morningness–Eveningness Scale for Children to determine their circadian typology wore a wrist activity device (Kronowise 3.0; Kronohealth SL) during school days and a weekend, participated. Circadian parameters, such as mesor, amplitude and acrophase, were estimated for skin temperature, light exposure and motor activity, as were sleep parameters, such as risetime, bedtime and social jetlag. The results indicated that during the weekend E‐type adolescents experienced later acrophases in temperature, light and activity than I‐type and M‐type adolescents did, whereas boys experienced earlier acrophases in temperature and activity. When school weekdays were compared with the weekend, there was a weekend delay in the acrophases of temperature (1:03), light exposure (2:03) and activity (3:15). The results obtained in this study provide external validity for applying the Morningness–Eveningness Scale for Children in the naturalistic context of high school while considering sex and type‐of‐day differences as important variables in chronopsychological studies.

## 1 INTRODUCTION

### 1.1 Morningness/eveningness during adolescence

Morningness/eveningness (M/E) is the most widely studied characteristic of the human circadian function. The M/E continuum reflects individual differences in the behavioural patterns of circadian rhythm, indicating an underlying trend towards advanced or delayed phase in a variety of circadian rhythms. Biological markers, such as body temperature, cortisol, melatonin and the sleep–wake rhythm, have been used to identify evening, intermediate and morning types (E‐, I‐ and M‐types, respectively; Duffy et al., 1999). Overall, previous findings suggest that the phase differences among individuals with extreme chronotypes can range from 2 to 12 hr, depending on the specific biological factors considered (Adan et al., 2012). The shift to eveningness during adolescence does not match conventional morning school schedules and, to meet school demands, most adolescents must try to function during the morning when they are in their non‐optimal moment of the day (Martin et al., 2016; Patte et al., 2017). Girls reported an earlier shift towards eveningness orientation in comparison to boys, probably due to their earlier pubertal development (Carskadon et al., 1993), although this tendency is moderated by psycho‐sociological factors, such as family relationships (Díaz‐Morales et al., 2014; Díaz‐Morales et al., 2023). Gradisar et al. (2011) estimated that eveningness impacts approximately 40% of adolescents, and compelling evidence suggests that eveningness during adolescence is related to emotional/behavioural problems, health‐impairing behaviours (e.g. sleep irregularity, eating disorders or substance abuse), depression (e.g. high mood seasonality or low positive affect), poor health‐related quality of life, and lower academic performance (Saxvig et al., 2021). On the other hand, evening adolescents have been described as right‐thinkers who tend to be creative and intuitive, with higher scores on intelligence than morning adolescents achieve (Díaz‐Morales & Escribano, 2014). The misalignment of the adolescent's rhythms (tendency towards eveningness) and the rhythms of the social environment (morning school hours) is the main hypothesis regarding the worst health indicators of adolescents. This hypothesis has received support from different studies in which E‐type adolescents who attended classes in the afternoon shift achieved similar academic performance to M‐type adolescents, reported lower social jetlag, and had a lower tendency towards depression (Goldin et al., 2020).

### 1.2 Morningness–Eveningness Scale for Children (MESC)

The MESC is one of the most frequently used self‐report questionnaires to assess M/E orientation or circadian preference in adolescents. The scale is an adaptation of the Composite Scale of Morningness (Smith et al., 1989) developed by Carskadon et al. (1993) for the adolescent population. Previous studies have reported good internal consistency, with Cronbach's alphas ranging from 0.70 to 0.82 for American, Australian, Croatian, Dutch, Hong Kong‐Chinese, Israeli, Italian, Mexican, Spanish, Taiwanese and Turkish adolescent samples; good test–retest reliability (*r*_s_ = 0.78–0.83); and external validity using various behavioural outcomes in adolescents (Tonetti et al., 2015). Regarding construct validity, a one‐factor measurement model and factorial invariance by age and sex have been found acceptable (Díaz‐Morales, 2015). With a sample of 5387 Spanish preadolescents and adolescents aged between 10 and 16 years, the 10/90th percentiles were established for 20/32 MESC values (25/75th percentiles for 23/28 MESC values). However, these cut‐off points have only been validated in Spanish adolescent samples with concurrent outcomes, such as sleep habits; health‐related quality of life; time‐of‐day effects on mood, anxiety and academic performance; cognitive abilities; daily fluctuations in attention at school; and autonomy and conflict with parents (for review, see Díaz‐Morales & Escribano, 2014). All these studies were performed on samples of adolescents aged 12–18 years who attended high school from 08:00 hours/08:30 hours to approximately 14:30 hours/15:30 hours. Therefore, it seems necessary to validate these cut‐off points with objective external variables.

### 1.3 Ambulatory circadian monitoring (ACM)

As a screening tool, questionnaires can be used to assess circadian preferences in adolescents to detect high risk. Most adult population M/E questionnaires have been validated via objective measures, such as temperature or actigraphy. However, the adolescent population questionnaires lack external validity. In the comprehensive review by Tonetti et al. (2015) concerning M/E measures in adolescents, it was recommended that future research should consider further validating the measures of the circadian preference of children and adolescents against objective‐external criteria.

To this end, authors have proposed validating such questionnaires via actigraphic measures. Progressive advances in ACM facilitate the measurement of different biological (e.g. temperature rhythm) and physical parameters (e.g. light exposure and motor activity) through ecological momentary methodology. This methodology offers several advantages, facilitating objective, non‐invasive recording of the sleep–wake patterns of individuals in their ecological context over long periods of time, and enabling analysis of the impact of physical and social synchronizers on circadian rhythmicity (Martinez‐Nicolas et al., 2019). Actigraphs are small, watch‐like devices that are usually placed on the non‐dominant wrist and contain motion detectors (accelerometers) to monitor and record movements. Although polysomnography (PSG) is considered the gold‐standard for sleep assessment, actigraphic data over several nights in the participant's natural environment can provide reliable estimates of sleep compared with PSG, which is typically performed over only 1 or 2 nights in a sleep laboratory (Fekedulegn et al., 2020).

In a study conducted by Santisteban et al. (2018), a sample of 115 participants aged 18–34 years was examined over 4–6 nights via the Munich Chronotype Questionnaire (MCTQ) and actigraphy. The results revealed high concordance (*p* = 0.51) and a strong correlation (*r* = 0.73) between the self‐reported midpoint of sleep and actigraphy. Additionally, Tonetti et al. (2024) validated the reduced Morningness–Eveningness Questionnaire for Children and Adolescents (rMEQ‐CA) via circadian motor activity assessed by actigraphy in 458 participants with a mean age of 15.7 years. They reported significant differences in the 24‐hr motor activity pattern between M‐ and E‐types. Similarly, Pracki et al. (2014) assessed 150 individuals aged 19–60 years via the Circadian Rhythm of Activity Questionnaire (KRAD), and reported that those classified as M‐types tended to fall asleep and wake up earlier, followed by I‐types, whereas individuals classified as E‐types exhibited later sleep schedules. Additionally, Kuula et al. (2022) reported that disruption in circadian rhythms was widely associated with psychiatric problems, such that a later sleep midpoint and a longer circadian period were associated with greater suicidality and depression. Girls presented a greater circadian amplitude in terms of skin temperature, and a greater circadian amplitude was observed in the majority of psychiatric problems.

Given the scarcity of studies in which circadian parameters are evaluated via ACM in the adolescent population, it would be useful to analyse circadian parameters, such as skin temperature, light exposure and motor activity. Body temperature has been the most extensively studied biological variable for differentiating extreme chronotypes (van Dongen, 1998). E‐types start their waking day at a lower body temperature than M‐types, and their temperature increases throughout the day, reaching its peak in the late afternoon. M‐types show a steeper rise in body temperature and reach their peak approximately 1 or 2 hr earlier than E‐types do. Skin temperature at the wrist has been proposed as a possible method to assess the circadian phase in free‐living conditions (Sarabia et al., 2008). It is a more comfortable measure for evaluating circadian rhythms in individuals and is also a good sleep marker for discriminating between chronotypes (Ortiz‐Tudela et al., 2014). Wrist temperature (T) follows the inverse pattern of body temperature: it increases at the beginning of the night, remains elevated during sleep, and decreases immediately after awakening.

Light exposure (L) is a highly relevant physical factor because chronotype is an expression of the phase angle of entrainment of the circadian system by the environmental light–dark cycle, and the degree of exposure to light depends on the activities of the individual (Martinez‐Nicolas et al., 2014). Work and school schedules may provide greater or lesser degrees of exposure to light. The few studies performed on adolescents attending either morning or afternoon school shifts have shown that participants are exposed to higher light intensities on school days than on free days, particularly in the morning (Anacleto et al., 2014; Estevan et al., 2022; Martin et al., 2016). Given that current actigraphy devices include photic sensors, monitoring L is a good indicator for comparing different samples in different contexts (Refinetti, 2019).

Motor activity (A) is also under circadian control and involved in the control of the sleep–wake cycle and numerous other physiological processes (Roveda et al., 2017). Considering that M‐types are characterized by earlier bedtime and rising time and better morning performance and that E‐types have later bedtime and rising time, motor activity is a useful behavioural index that the actigraph can be used to observe in ecological conditions (Tonetti, 2007). Individuals show variation in their preference for the daily timing of activity, with an earlier acrophase in M‐type individuals, whereas the other two circadian parameters of the activity rhythm (i.e. amplitude and mesor) show no differences among chronotypes (Lee et al., 2014).

The aim of the present study was to analyse the external validity of the MESC with objective and external criteria, such as T, L and A using the ACM (Madrid‐Navarro et al., 2019). It has been hypothesized that these three variables will follow a circadian variation with individual differences according to circadian preference such that M‐type adolescents will show an earlier acrophase than E‐type adolescents do, especially during the weekend, when they are unconstrained by morning school schedules.

## 2 METHODS

### 2.1 Participants

A total of 278 high school adolescents (154 boys and 124 girls), aged 12–13 years (12.43 ± 0.61), participated in the study. According to their MESC scores, 160 adolescents (73 boys and 87 girls; mean age = 12.19 ± 0.42 years) were selected to participate. Twenty‐two participants were eliminated because they did not wear the device long enough to collect valid physiological data. The final sample consisted of 138 adolescents, 57 boys and 81 girls. All the participants attended a public high school in the city of Madrid, Spain. None of the participants was receiving pharmacological treatment or taking hormonal contraception. The board of directors at the schools authorized the study after obtaining the parents' permission. Participation was voluntary, unpaid and anonymous. This study was performed according to international ethical standards (Portaluppi et al., 2010), and its procedures complied with the ethical standards outlined in the Helsinki 169 Declaration of 1975 (revised in 1983) and were previously approved by the institutional research committee at Psychology Faculty (Ref. 2020/21‐004).

#### 2.1.1 Instruments

##### Morningness–Eveningness Scale for Children (MESC)

The MESC was used to assess M/E orientation in adolescents. The scale consists of 10 questions written in a linguistic style that enables children and adolescents to understand them readily (Carskadon et al., 1993). Three questions have a response scale of five points (1–5), and seven questions have a response scale of four points (1–4). The outcome scores ranged from 10 (eveningness) to 43 (morningness). Previous studies have reported good internal consistency, good test–retest reliability, and external validity for various behavioural outcomes in adolescents, as previously described. In the present study, the reliability of the scale was 0.68 (Cronbach's alpha), and the range of corrected item–scale correlation coefficients was between 0.22 (Item 4) and 0.47 (Item 1).

##### Actigraphy

The participants wore the wrist‐worn actigraphy device Kronowise 3.0 (Kronohealth SL, Spain) on their non‐dominant hand for 6 days (from Wednesday to Monday). The actiwatch registers wrist skin temperature, triaxial motor acceleration, wrist posture and light exposure in three spectral bands (visible, blue from 460 to 490 nm, and infrared > 800 nm), and an electronic log (event marker) that could be used by the adolescents as an electronic diary was employed (Madrid‐Navarro et al., 2018). Wrist temperature (T), light exposure (L) and motor activity (A) were used in the present study.

#### 2.1.2 Sleep parameters

The exact moment at which the adolescent went to bed (bedtime, BT) and rose (risetime, RT) was recorded manually, according to the procedure described in Madrid‐Navarro et al. (2019). Bedtime was defined considering the drop in motor activity level, an absence of visible light and, if appropriate, the event marker of the device. Risetime was defined considering an increase in activity level, a decrease in skin temperature, an increase in light level above 1.0 μW cm^−2^ and, if applicable, an event marker. The time in bed and the sleep midpoint were subsequently calculated for the school days and the weekend. Social jetlag corrected by sleep debt (Jankowski, 2017) was computed as a measure of the misalignment between social and biological times (Wittmann et al., 2006). Additionally, the adolescents completed a paper–pencil diary entry at the end of the day before going to sleep, answering two questions: *What time did you get up today? What time did you go to bed yesterday?* The diary‐based sleep data were used as a validity check for the actigraphy‐based data.

### 2.2 Procedure and data analysis

The participants completed the MESC and answered demographic questions during the usual school schedule (08:30 hours–15:00 hours) in groups of approximately 25–30 adolescents under the supervision of the study's authors. The assessment sessions lasted approximately 30 min and were conducted in March 2022. E‐, I‐ and M‐types were selected using the 25/75 percentiles (equivalent to MESC values of 23/28) provided by Díaz‐Morales (2015). In the second phase, the selected adolescents donned the actigraph device for 6 days (Wednesday to Monday), wearing it on the non‐dominant wrist. Because device delivery and pick‐up occurred exclusively at their high school on Tuesdays, Tuesdays were not considered recording days in the analyses. All data collection took place during the same average photoperiod interval between May and June 2022. During this time interval, the photoperiod was approximately 14:25 hours, with sunrise occurring between 06:46 hours and 07:12 hours, and sunset occurring between 21:11 hours and 21:39 hours. The paper–pencil diary entry was completed at night before the participants slept. The participating adolescents had identical timetables and lived near their high school. None of the participants was aware of their own circadian type before the monitoring period.

The adolescents were instructed to wear the device for the majority of the monitoring period, except when performing intense sports activity and showering. They were also told that they should press the event marker on the device every time they went to bed and got up. Additionally, they were asked to take care to avoid covering the light sensor of the device with their sleeves. Twice during the week, an email was sent as a reminder to complete the diary entry and to encourage any participants having doubts. Using the dedicated software (Kronoware, version 10.0_L), the data were extracted from the device and recorded every 20 s. All the actograms were visually inspected prior to the subsequent data analyses. The procedure adopted to eliminate 22 participants, as mentioned in Section 2.1, was based on an analysis of the wrist temperature rhythm, which made it possible to detect when the device was removed. Days with more than 2 hr off‐wrist were excluded from the analyses. The data were averaged over consecutive 30‐min intervals to perform individual cosinor analysis on T, L and A throughout the day via the R package *Chronomics Analysis Toolkit* (CAT; Lee Gierke et al., 2013). To test the effects of chronotype and sex on the mesor, amplitude and acrophase circadian rhythm parameters of T, L and A, population cosinor analysis was used. Subsequently, the interaction between chronotype and sex with respect to the circadian parameters of T, L and A for both school days (Wednesday, Thursday, Friday and Monday) and the weekend were analysed. Finally, the circadian parameters of T, L and A were tested for differences between school days and weekend by repeated‐measures analysis of variance (rm‐ANOVA) considering within‐day effects (school days versus weekends) and between‐sex and chronotype effects. Partial eta‐squared ($\eta_{p}^{2}$) was used as a measure of effect size as follows. Effect sizes between 0.01 and 0.05 were considered low, those between 0.06 and 0.13 were considered moderate, and those above 0.14 were considered high (Cohen, 2016). All post‐hoc comparisons were Bonferroni corrected. The RStudio version 1.2.5033 program in R version 3.6.2 (R Core Team, 2019; R Studio Team, 2019) was used for the cosinor analysis. The program IBM SPSS Statistics version 25 (IBM Corp., 2017) was used to test the interactions between chronotype and sex as well as to test the differences between school days and weekend. The data were graphically represented via Microsoft Office Excel 2016. All the statistical tests were two‐tailed, and the type‐I error rate was set to 5% (*α* = 0.05).

## 3 RESULTS

The circadian parameters and M/E values were within the theoretical ranges. A total of 14,400 records were made for each adolescent. As expected, E‐types reported a lower average (M ± SD) MESC than I‐ and M‐types did (20.84 ± 2.85, 26.38 ± 1.14 and 31.29 ± 1.91, respectively; *F*_2,135_ = 279.42, *p* < 0.001, $\eta_{p}^{2}$ = 0.80). No differences were found according to sex. The percentage of boys and girls in each chronotype was as follows: E‐types: 71.2% girls, 28.8% boys; I‐types: 57.1% girls, 42.9% boys; and M‐types: 45.5% girls, 54.5% boys (*Χ* ^2^ = 6.55, *p* = 0.048).

A single cosinor analysis was performed on T, L and A, and circadian rhythm parameters (mesor, amplitude and acrophase) were calculated for each participant. The results revealed the presence of a statistically significant circadian rhythm (*p* < 0.001) for each participant. To examine the effects of sex and chronotype on each of the circadian rhythm parameters of the T, L and A patterns, we conducted a population‐wide cosinor analysis.

**FIGURE 1:**
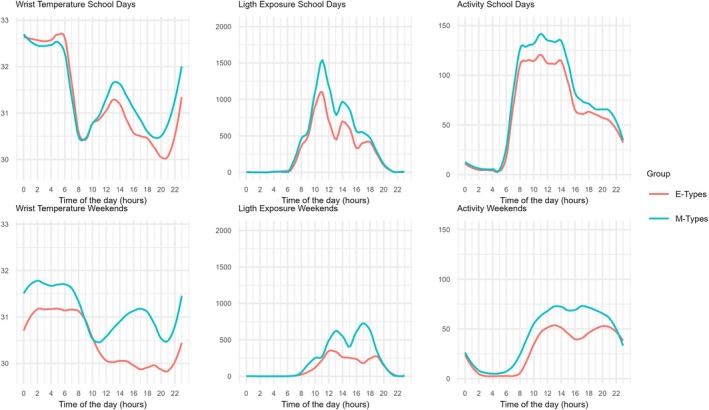
Wrist temperature, light exposure and activity 24‐hr patterns during school days and weekend according to circadian typology (intermediate types [I‐types] occupy an intermediate position between morning [M‐types] and evening types [E‐types], and are not represented for greater clarity).

The T pattern presented a plateau of high values at night and low values during the daytime (Figure 1). E‐types had a later acrophase on T than did I‐ and M‐types during the weekend. Pairwise comparisons revealed significant differences between the E‐type and M‐type groups (*F*_1,94_ = 6.05, *p* < 0.01), but not between the E‐type and I‐type groups (*F*_1,84_ = 2.64, *p* = 0.10). Boys exhibited a lower mesor and amplitude, earlier acrophase during the weekend, and a lower amplitude during school days (Table 1). None of the chronotype by sex interactions was significant.

**TABLE 1:** Rhythm metric analysis (population mean cosinor) for E‐, I‐, and M‐types during school days and weekend on wrist temperature (T)

	E‐types (*n* = 52)	I‐types (*n* = 42)	M‐types (*n* = 44)	All	*F*_2,135_	Boys (*n* = 57)	Girls (*n* = 81)	*F*_1,136_
All days								
Mesor	31.35 (0.42)	31.69 (0.42)	31.51 (0.41)	31.50 (0.23)	0.68	31.26 (0.38)	31.71 (0.30)	4.20*
Amplitude	0.99 (0.28)	1.01 (0.27)	0.67 (0.26)	0.89 (0.15)	1.71	0.54 (0.26)	1.14 (0.18)	14.76***
Acrophase	4:12 (3:46–4:44)	3:44 (3:07–4:19)	3:09 (2:11–4:03)	3:47 (3:26–4:08)	2.62*	3:04 (2:00–4:00)	4:02 (3:42–4:22)	4.18*
School days								
Mesor	31.55 (0.41)	31.77 (0.40)	31.62 (0.41)	31.64 (0.23)	0.30	31.43 (0.38)	31.79 (0.28)	2.27
Amplitude	1.06 (0.31)	1.07 (0.29)	0.75 (0.29)	0.96 (0.17)	1.36	0.63 (0.25)	1.19 (0.22)	10.93***
Acrophase	3:47 (4:36–6:13)	3:23 (3:44–5:32)	3:07 (0:28–4:53)	3:29 (3:05–3:54)	0.85	3:24 (2:18–4:41)	3:31 (3:10–3:53)	0.05
Weekend								
Mesor	30.94 (0.48)	31.53 (0.51)	31.29 (0.51)	31.23 (0.28)	1.42	30.77 (0.44)	31.55 (0.36)	7.15***
Amplitude	0.90 (0.36)	0.89 (0.39)	0.50 (0.32)	0.75 (0.21)	1.41	0.38 (0.29)	1.09 (0.23)	11.84***
Acrophase	5:11 (4:24–6:05)	4:34 (3:32–5:24)	3:03 (0:02–4:40)	4:32 (3:54–5:05)	3.17*	1:48 (21:19–3:35)	5:08 (4:36–5:40)	11.66***

E‐type, evening; I‐type, intermediate; M‐type, morning.

* *p* < 0.05.

*** *p* < 0.001.

We subsequently compared the differences between school days and weekend. Compared with school days, there was a decrease in the mesor of T during the weekend (*F*_1,132_ = 25.28, *p* < 0.001). This decrease was greater in boys than in girls (boys = 0.66 and girls: 0.24; *F*_1,132_ = 7.96, *p* < 0.01). There was no difference in the amplitude of T, but there was a delay of 1:03 in the acrophase of T during the weekend (*F*_1,132_ = 14.58, *p* < 0.001, $\eta_{p}^{2}$ = 0.10; Table 1).

The L pattern presented the lowest values at night and the highest values during the day (Figure 1). E‐type adolescents were exposed to less light (mesor) and had lower amplitude during the school days. For both the mesor and the amplitude of L, pairwise comparisons revealed significant differences between the E‐type and M‐type groups (*F*_1,94_ = 4.86, *p* < 0.05; *F*_1,94_ = 6.07, *p* < 0.05), but not between the E‐type and I‐type groups (*F*_1,84_ = 1.85, *p* = 0.17; *F*_1,84_ = 2.51, *p* < 0.11). E‐types had a later acrophase on L compared with I‐ and M‐types during the weekend. Pairwise comparisons revealed significant differences between the E‐type and M‐type groups (*F*_1,94_ = 4.05, *p* < 0.05), but not between the E‐type and I‐type groups (*F*_1,84_ = 1.74, *p* = 0.23). Boys exhibited a higher mesor and amplitude on L during the school days (Table 2).

**TABLE 2:** Rhythm metric analysis (population mean cosinor) for E‐, I‐ and M‐types during school days and weekend on light exposure (L)

	E‐types (*n* = 52)	I‐types (*n* = 42)	M‐types (*n* = 44)	All	*F*_2,135_	Boys (*n* = 57)	Girls (*n* = 81)	*F*_1,136_
All days								
Mesor	258.19 (52.35)	286.60 (73.03)	359.59 (79.33)	299.17 (38.69)	2.46*	346.65 (75.15)	265.75 (39.29)	4.24*
Amplitude	345.42 (66.17)	384.60 (95.50)	503.31 (116.54)	407.18 (53.11)	3.24*	480.71 (104.39)	355.61 (51.97)	5.42*
Acrophase	13:32 (13:08–13:54)	13:25 (13:02–13:47)	13:04 (12:36–13:30)	13:22 (13:08–13:35)	1.85	13:17 (12:54–13:36)	13:22 (13:08–13:44)	0.57
School days								
Mesor	318.35 (59.72)	338.62 (67.22)	419.59 (80.87)	356.80 (39.54)	2.44*	412.36 (72.65)	317.70 (43.27)	5.61**
Amplitude	433.71 (59.72)	468.98 (67.22)	603.24 (80.87)	498.05 (55.69)	3.49*	587.99 (105.32)	434.84 (57.38)	7.50**
Acrophase	13:04 (12:40–13:29)	13:02 (12:42–13:22)	12:42 (12:13–13:10)	12:57 (12:44–13:10)	0.96	12:54 (12:33–13:14)	13:00 (12:42–13:18)	0.21
Weekend								
Mesor	140.20 (53.68)	183.48 (126.23)	240.86 (113.90)	185.47 (55.51)	1.11	218.24 (112.01)	162.40 (54.26)	0.95
Amplitude	191.75 (72.88)	238.90 (153.41)	346.19 (170.41)	254.71 (75.92)	1.44	294.34 (149.66)	227.51 (78.97)	0.73
Acrophase	15:16 (14:37–15:58)	15:02 (13:42–16:18)	14:37 (13:34–17:06)	15:00 (14:26–15:40)	2.05*	14:47 (13:48–16:06)	15:12 (14:32–15:56)	0.50

E‐type, evening; I‐type, intermediate; M‐type, morning.

* *p* < 0.05.

** *p* < 0.01.

*** *p* < 0.001.

Compared with the school days, there was a decrease in the mesor and amplitude of L during the weekend (*F*_1,132_ = 38.71, *p* < 0.001 and *F*_1,132_ = 33.08, *p* < 0.001, respectively). Additionally, there was a delay of 2:03 in the acrophase of L during the weekend (*F*_1,132_ = 88.83, *p* < 0.001, $\eta_{p}^{2}$ = 0.40; Table 2).

The A pattern presented the lowest values at night and the highest values during the day (Figure 1). E‐types had a later acrophase on A compared with I‐ and M‐types during the weekend. Pairwise comparisons indicated significant differences between E‐ and M‐types (*F*_1,94_ = 5.05, *p* < 0.01), but not between I‐ and M‐types (*F*_1,84_ = 0.84, *p* = 0.45). Boys exhibited an earlier acrophase on weekends (Table 3). Additionally, boys had an earlier acrophase on A during the school days, but this difference was marginally significant (*p* = 0.07).

**TABLE 3:** Rhythm metric analysis (population mean cosinor) for E‐, I‐ and M‐types during school days and weekend on Activity (A)

	E‐types (*n* = 52)	I‐types (*n* = 42)	M‐types (*n* = 44)	All	*F*_2,135_	Boys (*n* = 57)	Girls (*n* = 81)	*F*_1,136_
All days								
Mesor	56.75 (9.36)	63.01 (9.83)	65.50 (9.18)	61.44 (5.37)	0.96	59.80 (8.98)	62.60 (6.78)	0.25
Amplitude	44.78 (7.84)	49.60 (8.03)	54.53 (8.37)	49.34 (4.59)	1.53	52.14 (7.76)	47.69 (5.78)	0.89
Acrophase	14:04 (13:38–14:26)	13:53 (13:28–14:15)	13:50 (13:28–14:11)	13:56 (13:42–14:08)	0.41	13:33 (13:13–13:52)	14:13 (13:56–14:28	9.94***
School days								
Mesor	66.92 (10.19)	71.56 (10.09)	75.50 (9.7)	71.07 (5.71)	0.76	71.47 (9.67)	70.79 (7.12)	0.01
Amplitude	56.95 (8.73)	60.53 (8.46)	67.05 (9.74)	61.25 (5.11)	1.34	66.10 (8.85)	57.95 (6.21)	2.432
Acrophase	13:20 (12:57–13:40)	13:12 (12:48–13:34)	13:10 (12:51–13:29)	13:14 (13:02–13:26)	0.25	13:02 (12:44–13:20)	13:24 (13:08–13:38)	3.23^+^
Weekend								
Mesor	36.80 (8.58)	45.86 (11.13)	45.44 (10.72)	42.31 (5.69)	1.11	36.63 (9.53)	46.31 (7.05)	2.77
Amplitude	31.33 (8.83)	36.73 (10.57)	39.39 (10.68)	35.43 (5.62)	0.72	31.27 (9.12)	38.85 (7.22)	1.74
Acrophase	17:05 (16:12–17:42)	16:18 (15:36–16:58)	16:10 (15:40–16:53)	16:30 (16:07–16:53)	2.17*	15:51 (15:13–16:29)	16:52 (16:24–17:20)	6.15*

E‐type, evening; I‐type, intermediate; M‐type, morning.

* *p* < 0.05.

+ *p* < 0.07.

*** *p* < 0.001.

Compared with the school days, there was a decrease in the mesor and amplitude of A during the weekend (*F*_1,132_ = 189.46, *p* < 0.001 and *F*_1,132_ = 91.27, *p* < 0.001, respectively). Regarding the mesor, this decrease was greater in boys than in girls (boys = 34.84; girls = 24.48; *F*_1,132_ = 6.94, *p* < 0.001), as was the decrease in amplitude (boys = 34.83; girls = 13.1; *F*_1,132_ = 12.20, *p* < 0.001). Additionally, there was a delay of 3:15 in the acrophase of A during the weekend (*F*_1,132_ = 13.89, *p* < 0.001, $\eta_{p}^{2}$ = 0.095; Table 2).

With respect to the sleep parameters (reported in hours and minutes format, M ± SD) derived from motor activity (A), the analysis of variance of repeated‐measures revealed a within‐effect of the type of day (school day versus weekend) in risetime (*F*_1,130_ = 455.1, *p* < 0.001, $\eta_{p}^{2}$ = 0.778), which was later during the weekend (09:21 hours ± 1:24) than during school days (07:22 hours ± 0:27); an effect of the interaction type of day*chronotype (*F*_2,130_ = 5.27, *p* < 0.01, $\eta_{p}^{2}$ = 0.075), whereby the delay in risetime was greater for E‐types (from 07:29 hours ± 0:04 to 10:28 hours ± 0:12) than for I‐types (07:18 hours ± 0:04 to 09:34 hours ± 0:12) and M‐types (07:21 hours ± 0:03 to 09:29 hours ± 0:12); and a marginally significant effect in risetime of the type of day*sex interaction (*F*_1,130_ = 3.55, *p* = 0.062, $\eta_{p}^{2}$ = 0.027), in which the delay was greater for girls (07:20 hours ± 0:03 to 10:01 hours ± 0:09) than for boys (07:25 hours ± 0:03 to 09:37 hours ± 0:10).

There was also a delay in bedtime when the type of day, i.e. whether a school days (22:54 hours ± 0:57) or weekend (23:38 hours ± 1:13), was considered (*F*_1,130_ = 49.68, *p* < 0.001, $\eta_{p}^{2}$ = 0.27). With respect to sleep length, during school days, sleep duration was shorter (08:15 hours ± 0:34) than during the weekend (10:07 hours ± 1:25; *F*_1,130_ = 138.10, *p* < 0.001, $\eta_{p}^{2}$ = 0.51); the type of day*sex interaction indicated a greater increase in the duration of sleep length for girls (08:15 hours ± 0:06 to 10:22 hours ± 0:11) than for boys (08:22 hours ± 0:08 to 09:34 hours ± 0:07; *F*_1,130_ = 6.43, *p* < 0.05, $\eta_{p}^{2}$ = 0.47); and the type of day*chronotype interaction revealed a greater increase in sleep length for E‐types (from 08:22 hours ± 0:08 to 10:39 hours ± 0:15) than for I‐types (from 08:17 hours ± 0:08 to 09:43 hours ± 0:15) and M‐types (from 08:37 hours ± 0:08 to 10:07 hours ± 0:15; *F*_1,130_ = 3.27, *p* < 0.05, $\eta_{p}^{2}$ = 0.48). Finally, regarding the sleep midpoint, the effect of the type of day indicated a later sleep midpoint on the weekend (04:45 hours ± 0:59) than on school days (03:09 hours ± 0:34; *F*_1,130_ = 404.48, *p* < 0.001, $\eta_{p}^{2}$ = 0.75), and the type of day*circadian type interaction indicated a later sleep midpoint between school days and the weekend for E‐types (from 03:18 hours ± 0:05 to 05:08 hours ± 0:09) than for I‐types (from 03:09 hours ± 0:04 to 04:42 hours ± 0:09) and M‐types (from 03:03 hours ± 0:04 to 04:25 hours ± 0:08; *F*_1,130_ = 3.03, *p* < 0.05, $\eta_{p}^{2}$ = 0.47).

With respect to the relationships between MESC scores and sleep parameters, the Pearson correlation coefficients (*p* < 0.05) indicated that greater morningness was associated with an earlier bedtime during school days (*r* = −0.19), an earlier bedtime on weekends (*r* = −0.38), shorter sleep duration during weekends (*r* = −0.24), an earlier sleep midpoint both during school days and weekends (*r* = −0.19 and *r* = −0.33, respectively) and reduced social jet lag (*r* = −0.32).

## 4 DISCUSSION

The results obtained in this study provide external validity for the MESC, which enabled us to distinguish among morning, intermediate and evening adolescents in terms of the circadian parameters of temperature, light exposure and motor activity. To our knowledge, the MESC had not yet been validated for these objective variables in this age group while also considering sex and type of day (school days and weekend), two variables that are highly relevant for the adolescent population (Sen & Spruyt, 2020; Tonetti et al., 2015).

Given that the wrist body temperature rhythm has an inverse phase relationship with core body temperature (beginning to increase in association with bedtime and decreasing sharply after awakening; Sarabia et al., 2008), the wrist temperature results align with expectations, revealing a 2‐hr earlier acrophase for morning‐type adolescents during the weekend (Montaruli et al., 2021). It has also been reported that boys have earlier wrist temperature acrophases, whereas girls had higher mesor and amplitude values. During school days, girls exhibited greater amplitudes. These findings suggest that weekend circadian preference differences in acrophases are more pronounced than those on school days. The increased circadian amplitude of temperature in girls could be related to the greater distinctiveness found in previous studies in both adults (Carciofo, 2020) and adolescents (Puig‐Navarro & Díaz‐Morales, 2024). The differences found in the circadian temperature parameters obtained during school days and weekends also support the well‐documented phase delay in sleep habits during the weekend (i.e. longer duration of nocturnal sleep, delayed sleep midpoint and greater social jetlag; Lang et al., 2022; Zhang et al., 2020).

With respect to light exposure, E‐types had lower light exposure, and the amplitude of the light pattern was smaller during school days. Additionally, girls had lower light exposure, and the amplitude of the light pattern was smaller. During the weekend, M‐types had earlier acrophases than E‐types did. Comparing school days with the weekend, there was a decrease in the mesor and amplitude of the L pattern and a delay of 2:03 hours of exposure to light. These results corroborate previous studies in which average light exposure was lower on both free and vacation days than on school days in both adolescents (Estevan et al., 2022) and adults (Crowley et al., 2015; Zerbini et al., 2021). However, Read et al. (2014) reported higher light intensity on weekends than on weekdays, likely because of the increased physical activity observed on weekends; thus, more research is needed to explore differences in daytime activity patterns in relation to light exposure considering the type of day.

With respect to physical activity, differences appeared according to circadian typology during the weekend, with such activity occurring nearly 1 hr earlier in M‐types and boys, who were more active in the early afternoon than were E‐types and girls, whose activity peaked in the late afternoon. Montaruli et al. (2021) and Paciello et al. (2022) reported similar rest–activity acrophase differences, and Plekhanova et al. (2023) reported that, compared with M‐types, E‐types exhibited more sedentary time (10 min per day) and had lower overall physical activity (i.e. average acceleration).

The differences in sleep parameters evaluated via actigraphy align with those reported in previous studies. Vitale et al. (2015) reported that M‐types went to bed approximately 2 hr earlier than E‐types did on both weekdays and weekends, and that E‐types woke up significantly later than M‐types did. Similarly, Lee et al. (2014) reported significant differences in bedtimes, wake times and mid‐sleep timing on both workdays and free days, with E‐types going to bed and waking up later than M‐types and having significantly longer sleep durations on free days.

Finally, this study has several limitations. Ambulatory circadian assessment of skin temperature, light exposure and motor activity in free‐living conditions can be significantly affected by variables such as fluctuating environmental temperatures, dietary habits and sport activities. Physical variables, such as height and weight, were not controlled for, nor was pubertal development taken into account. However, our study has several strengths. It was conducted within the natural context of adolescent life. The sample consisted of a substantial number of adolescents, encompassing both sexes and circadian preferences, allowing for a comprehensive analysis of the interactions between these variables. Additionally, the 6‐day recording period facilitated the analysis of the differences in circadian parameters between weekdays and weekends. Finally, normative data are provided on skin temperature, light exposure and motor activity in the adolescent population that have enabled the validation of one of the most commonly used questionnaires for the evaluation of circadian preferences in adolescents.

## AUTHOR CONTRIBUTIONS

**Yaiza Puig‐Navarro:** Investigation; writing – review and editing; visualization; methodology; data curation; software; formal analysis. **Juan F. Díaz‐Morales:** Conceptualization; funding acquisition; writing – original draft; writing – review and editing; methodology; project administration; supervision; resources; validation.

## FUNDING INFORMATION

This study was supported by the Plan Estatal de Investigación Científica, Técnica y de Innovación (2021–2023), Ministerio de Ciencia e Innovación, Spain (JFDM; Ref. PID2020‐116600RB‐I00). The funding did not influence any content or choices relating to this study.

## CONFLICT OF INTEREST STATEMENT

The authors assert they have no conflict of interest with any organization or entity with any financial interest or non‐financial interest in the subject matter or materials discussed in this manuscript.

## Data Availability

The data that support the findings of this study are available from the corresponding author upon reasonable request.
